# The Formin mDia1 Regulates Acute Lymphoblastic Leukemia Engraftment, Migration, and Progression *in vivo*

**DOI:** 10.3389/fonc.2018.00389

**Published:** 2018-09-20

**Authors:** Scott B. Thompson, Eric J. Wigton, Sai Harsha Krovi, Jeffrey W. Chung, Robert A. Long, Jordan Jacobelli

**Affiliations:** ^1^Department of Biomedical Research, National Jewish Health, Denver, CO, United States; ^2^Department of Immunology and Microbiology, University of Colorado School of Medicine, Aurora, CO, United States

**Keywords:** leukemia engraftment, transendothelial migration, cytoskeleton, formins, mDia1, Diaph1

## Abstract

Leukemias typically arise in the bone marrow and then spread to the blood and into other tissues. To disseminate into tissues, leukemia cells migrate into the blood stream and then exit the circulation by migrating across vascular endothelial barriers. Formin proteins regulate cytoskeletal remodeling and cell migration of normal and malignant cells. The Formin mDia1 is highly expressed in transformed lymphocytes and regulates lymphocyte migration. However, the role of mDia1 in regulating leukemia progression *in vivo* is unknown. Here, we investigated how mDia1 mediates the ability of leukemia cells to migrate and disseminate *in vivo*. For these studies, we used a mouse model of Bcr-Abl pre-B cell acute lymphoblastic leukemia. Our data showed that mDia1-deficient leukemia cells have reduced chemotaxis and ability to complete transendothelial migration *in vitro*. *In vivo*, mDia1 deficiency reduced the ability of leukemia cells to engraft in recipient mice. Furthermore, leukemia dissemination to various tissues and leukemia progression were inhibited by mDia1 depletion. Finally, mDia1 depletion in leukemia cells resulted in prolonged survival of recipient mice in a leukemia transfer model. Overall, our data show that the Formin mDia1 mediates leukemia cell migration, and drives leukemia engraftment and progression *in vivo*, suggesting that targeting mDia1 could provide a new method for treatment of leukemia.

## Introduction

Acute lymphoblastic leukemias (ALL) typically arise from the unrestrained proliferation of transformed leukocyte precursors in the bone marrow. This type of leukemia is the most common form of cancer in children (1). Leukemia cells eventually reach the blood circulation and from there can disseminate to various tissues. Leukemia dissemination is associated with a poor prognosis. The ability of leukemia cells to disseminate to various tissues relies in part on their ability to migrate out of the blood stream and infiltrate tissues. A necessary step for the exit of leukemia cells from the blood circulation is migration through the endothelial cell wall of blood vessels, a process referred to as transendothelial migration.

In the case of tumors of hematological origin, such as leukemias and lymphomas, many of the adhesion and homing molecules required for the transendothelial migration process are the same as those used by non-transformed lymphocytes (2, 3). This suggests that the basic mechanisms driving lymphocyte transendothelial migration and tissue infiltration are likely shared between normal and transformed cells. During transendothelial migration, leukocytes undergo a multi-step process by which they initially roll on the endothelial vascular wall, in a selectin-dependent manner, adhere to the endothelium through a chemokine-induced integrin-mediated process, and finally crawl and squeeze through the endothelial barrier (4, 5). During the various phases of transendothelial migration, leukocytes change shape, and extend membrane protrusions at their leading edge (6), which are processes that require actin network remodeling and force generation by cytoskeletal motors (7–9). Furthermore, data have shown that genes involved in cytoskeletal remodeling and cell migration play an important role in lymphoma progression *in vivo* (10). We recently reported that the cytoskeletal motor protein Myosin-IIA is required for leukemia migration, progression and entry into the Central Nervous System (11). However, the role of specific cytoskeletal effector proteins in leukemia migration and progression *in vivo* is largely unknown.

Among the cytoskeletal effectors that regulate actin dynamics in lymphocytes are members of the Formin family, of which Diaphanous-related formin-1 (mDia1, Diaph1) and Formin-like-1 (FMNL1) are the main Formin proteins expressed in lymphocytes (12, 13). Formins promote the polymerization of linear actin filaments by processively adding actin monomers to generate and elongate actin filaments (14, 15). In addition to actin nucleation and polymerization, Formins also regulate microtubules and have been shown to play a role in various cellular processes including cell division, polarization, adhesion, and migration (14, 16). Furthermore, Formins have also been implicated in mediating the migration and invasion of malignant cells (17–19).

In leukocytes, Formins regulate motility, trafficking and activation (20–23). In response to various stimuli, including chemokine stimulation, and downstream of Rho-GTPase activation, Formins reorganize the actin cytoskeleton, a process required for motility and transendothelial migration (6, 7). Specifically, mDia1 is highly expressed in transformed lymphocytes and regulates T lymphocyte migration *in vitro* (24). *In vivo*, mDia1 plays an important role in T lymphocyte development, migration and trafficking (20, 21). This Formin has also been shown to play a role in T cell actin polymerization, activation, and proliferation (20, 21, 24). Furthermore, the Formin FMNL1 is overexpressed in lymphoid malignancies (25, 26) and recently FMNL1 has been implicated in regulating leukemia proliferation and migration (27).

Based on the role of mDia1 in cytoskeletal rearrangements and lymphocyte motility, as well as the reported role of FMNL1 in leukemia migration (27), we sought to determine the possible role of mDia1 in leukemia cell migration, dissemination, and progression *in vivo*. We found that mDia1 deficiency in leukemia cells reduced their chemotaxis and ability to complete transendothelial migration. We also discovered a role for mDia1 in leukemia extravasation and engraftment *in vivo*. These migration defects resulted in poor engraftment, slower progression of mDia1-deficient leukemia, and ultimately prolonged survival in a leukemia transfer model. Our data suggest that either mDia1 or the upstream pathways that regulate its function are possible therapeutic targets for the treatment of leukemia dissemination and progression.

## Materials and methods

### Ethics statement

This study and protocol were reviewed and approved by the Institutional Animal Care and Use Committee at National Jewish Health, and all efforts were made to minimize mouse suffering.

### B-ALL cell transduction and shRNA constructs

The Arf-negative BCR-ABL-positive Acute Lymphoblastic Leukemia cells (B-ALL) were generously provided by Dr. James DeGregori (University of Colorado, School of Medicine), and established by Sherr and colleagues (28). The B-ALL cells were retrovirally transduced using pSiren-RetroQ vectors to express mDia1 specific shRNAs (mDia1 KD B-ALL, sequence #1 GCGCAGAATCTCTCAATCTTT; sequence #2 GGACATCTCAGACGAGCAATT) or a control non-silencing shRNA (Control B-ALL, sequence TCTATAGAACCCTCAATAT). The pSiren vectors co-expressed ZsGreen or DsRed as a marker and the B-ALL transduced cells were sorted based on green or red fluorescence. Sorted cells were cultured *in vitro* for no more than 6 weeks and knock-down (KD) was monitored routinely by western blot and verified to be at least 85% compared to control B-ALL cell mDia1 expression. Every 6 weeks of culture transduced B-ALL cells were refreshed using cryogenically stored aliquots.

### Western blot analysis

Protein levels were determined using an anti-mDia1 rabbit polyclonal antibody (ECM Biosciences) or anti-FMNL1 rabbit polyclonal antibody (Sigma). Mouse anti-tubulin (Sigma) was used as a loading control. Antibody staining was detected using the Odyssey near-infrared imaging system (Li-cor Biosciences) with IRDye-680 or-800 secondary antibodies.

### Apoptosis assay

The steady-state frequency of apoptotic B-ALL leukemia cells was measured by staining with APC-Annexin V (Becton Dickinson). Control and mDia1 KD B-ALL cells cultured for 48 h at 37°C were stained with Annexin V and analyzed by flow cytometry using an LSR Fortessa (Becton Dickinson). Data was analyzed using Flowjo (Flowjo) and the frequency of apoptotic cells was determined by measuring the Annexin V positive population.

### *In vitro* cell growth curves

B-ALL leukemia cells were grown in RPMI 1640 (MediaTech), with 10% FBS (Hyclone) 5 μM BME (Thermo Fisher), Penicillin, Streptomycin, and L-glutamine (Thermo Fisher). For growth curve determination, B-ALL cells were plated at 5 × 10^5^/mL and then diluted every 2 days by a 1:4 factor. Cell numbers were determined by hemocytometer using Trypan Blue (Sigma) for dead cell exclusion. B-ALL proliferation was monitored for 6 days and growth curves were determined by compounding cell numbers over the growth period.

### Transwell migration assay

Control or mDia1 KD B-ALL cells were resuspended in RPMI + 2% BSA +10 mM HEPES and added to 5 μm pore transwell inserts (Corning). The bottom chambers of a 24 well transwell plate contained the same RPMI + 2% BSA +10 mM HEPES with or without 1 μg/mL of CXCL12/SDF1-α (Peprotech). As a standard to calculate the percentage of migrated cells, 4 × 10^5^ cells (20% of input cells added to the transwell inserts) were plated into bottom wells with no transwell. The plate was incubated for 2 h at 37°C and then B-ALL cells were harvested from the bottom wells and analyzed by flow cytometry using counting beads (Thermo Fisher) for standardization.

### Transendothelial migration under flow assay

Forty-eight hours prior to the assay bEnd.3 endothelial cells were plated in tissue culture treated μ-Slide VI 0.4 flow chambers (ibidi). Twenty-four hours later, the endothelial monolayer was treated with 40 ng/mL TNF-1α (Peprotech), which upregulates expression on the bEnd.3 endothelial cells of adhesion molecules (such as ICAM-1 and VCAM-1) needed to support leukocyte TEM. Then 30–45 min prior to the assay the endothelial cells were treated with 1 μg/mL CXCL12, which promotes the rolling and adhesion of leukocytes on the endothelial cells. For the transendothelial assay, using a syringe pump, control, or mDia1 KD B-ALL cells (at 2 × 10^6^ cells/mL) were flowed onto the treated endothelial monolayer at 0.25 dyne/cm^2^ for 5 min (accumulation phase), and then the flow rate was increased to 2 dyne/cm^2^ (approximate physiological shear flow). Phase contrast and fluorescent images were acquired every 15–25 s using a 20X Phase-2 objective for 30 min long time-lapses using a Spinning Disk confocal microscope with environmental control (Intelligent Imaging Innovations) and Slidebook imaging software (Intelligent Imaging Innovations). Using similar criteria as previously described (11, 29), a cell was scored as having undergone transendothelial when it lost its white phase ring in a step-wise process during the course of the time-lapse.

### *In vivo* short-term B-ALL co-adoptive transfer

Control or mDIa1 KD B-ALL cells were stained for 15 min at 37°C in HBSS (MediaTech) with either 1 μM Violet Proliferation Dye 450 (Becton Dickinson) or 5 μM Cell Proliferation Dye eFluor670 (eBioscience). Dye-labeled cells (2.5 × 10^6^) were then transferred at a 1:1 ratio in 8–16 week old C57BL/6 CD45.1+ recipient mice (Charles River) by tail vein injection and 24 h later the recipient mice were euthanized and organs were harvested for flow cytometry analysis as described below. To rule-out effects of the different dyes used for labeling, between experimental repeats the dye used to label each population was swapped.

### Long-term *in vivo* leukemia dissemination and survival assays

Control or mDia1 KD B-ALL cells were adoptively transferred by tail vein injection into cohorts of 5–6 age-matched 8–12 week old CD45.1+ male recipient mice (5 × 10^4^ cells/mouse). Mice were monitored daily, and mice showing signs of morbidity (hunched position, lethargy, ruffled fur) and/or body weight loss >15% from original weight were euthanized for humane reasons. For time-course experiments, randomly chosen pairs of mice (1 control and 1 mDia1 recipient) were selected every 3 days (from day 3 to 9 post-transfer). The recipient mice were euthanized and organs harvested for flow cytometry analysis as described below.

### Tissue processing and analysis

All mice were euthanized with CO_2_ according to our humane institutional IACUC procedures. After euthanasia, blood was extracted via cardiocentesis and mice were subsequently perfused with saline. For experiments with intravascular cell labeling, 4 min prior to euthanasia mice were injected via tail vein with 3 μg of anti-CD19-APC (Biolegend, Clone 6D5). Bone marrow, spleen, brain, and spinal cord were harvested and mechanically dissociated, the liver was digested by collagenase D (0.786 Wunsch U/mL and DNase I 250 ug/ml [Roche]). Spleen and blood were treated with 175 mM ammonium chloride (Sigma) for 5 min at room temperature to lyse red blood cells. Lymphocytes from brain and spinal cord samples were isolated using a 70/30% Percoll gradient (Sigma). Lymphocytes from liver samples were isolated using Histopaque 1119 (Sigma). To identify transferred leukemia cells, samples were stained with anti-CD45.1 PacBlue (Biolegend, Clone A20), except for dye labeled B-ALL samples, and analyzed on a Cyan ADP flow cytometer (Beckman Coulter). Transferred B-ALL cells were identified as CD45.1-negative and fluorescent marker-positive. For the staining procedures, to prevent non-antigen specific binding of IgG antibodies to Fcγ receptors, prior to staining with antigen specific labeled antibodies, we incubated the samples with blocking anti-CD16/CD32 antibodies (BioXcell). All flow data was analyzed on Flowjo.

### Statistical analysis

Prism software (Graphpad) was used to create graphs and perform statistical analyses. To determine statistical significance, for single comparisons the Student's paired *t*-test was used, for multiple comparisons ANOVA was performed followed by post-tests, and for two independent variables analysis a 2-way ANOVA was used followed by post-tests. Survival curve data was analyzed using the log-rank test.

## Results

### mDia1 knock-down does not increase leukemia cell apoptosis or reduce proliferation

**Figure 1:**
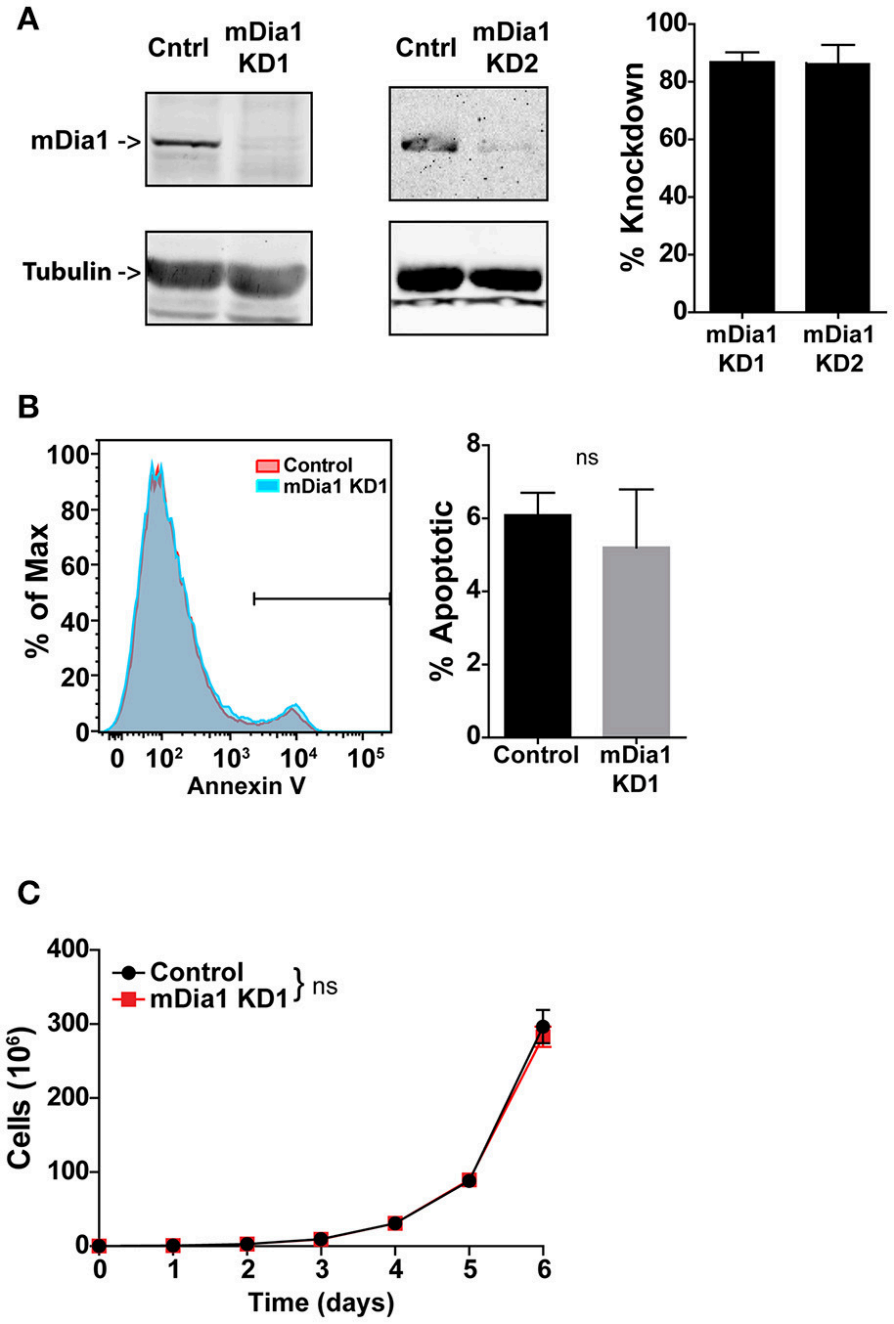
mDia1 knock-down does not affect B-ALL cell viability and proliferation. B-ALL cells were transduced with two independent shRNA constructs targeting mDia1 (mDia1 KD1 and mDia1 KD2) or with a control shRNA. The transduced cells were sorted based on the co-expression of a fluorescent marker (ZsGreen or DsRed) on the shRNA vector. **(A)** mDia1 is depleted in B-ALL cells transduced with mDia1-specifc shRNAs. Left panels, Western blot analysis of cell lysates from control and mDia1 knock-down (KD) cells. Tubulin staining is shown for normalization purposes. Right panel, quantification of KD levels in the two mDia1 KD cell lines. **(B)** B-ALL cell apoptosis is not increased in mDia1 KD cells. Left, representative flow cytometry staining for Annexin V of control and mDia1 KD cells. Right, quantification of the frequency of apoptotic cells. **(C)** mDia1 KD does not impair B-ALL proliferation. *In vitro* proliferation of B-ALL cells over the course of 6 days. Data in **(A,C)** are the average of at least 4 independent experiments; data in **(B)** are the average of at least 3 independent experiments. Error bars are the SEM.

For these studies we employed a murine model of pre-B-cell ALL (B-ALL). This leukemia cell line was developed by transducing the Bcr-Abl fusion protein into bone marrow cells from Arf-negative C57BL/6 mice (28). This adoptive transfer leukemia model is highly aggressive and typically induces overt leukemia in syngeneic non-irradiated mice within 2–3 weeks of transfer (28, 30, 31). We used a short-hairpin RNA (shRNA) transduction approach to knock-down mDia1 protein expression in this B-ALL line to investigate the role of mDia1 in leukemia cell migration, engraftment and progression. B-ALL cells were stably transduced with retroviral vectors encoding for a control shRNA or, to ensure specificity, two different shRNA sequences targeting the mDia1 mRNA. For shRNA delivery we used a retroviral vector that co-expresses a fluorescent marker (ZsGreen or DsRed) to sort and purify the transduced leukemia cells. Our results showed that in sorted B-ALL cells we could routinely deplete mDia1 protein with an average knock-down (KD) level of ~86% for each of the two targeting shRNAs compared to B-ALL cells transduced with control shRNA (Figure 1A). We next investigated if as a compensatory mechanism, the other main Formin expressed in lymphocytes, FMNL1, was upregulated in the mDia1 KD cells. Our western blot analysis did not show any major change in FMNL1 expression between control and mDia1 KD cells (Supplemental Figure 1A).

Formin proteins function in cytokinesis and, potentially, cell division (32, 33, 34). Therefore, we analyzed if mDia1 depletion altered B-ALL cell viability and growth. Using a flow cytometry-based assay we measured B-ALL cell apoptosis and found no significant difference in the number of Annexin-V^+^ apoptotic cells comparing control and either of the two mDia1 KD cells (Figure 1B and Supplemental Figure 1B). We then determined if mDia1 KD affected B-ALL cell proliferation. For these experiments we compared control and mDia1 KD B-ALL growth over a period of 6 days and found no significant difference in control and mDia1 KD B-ALL cell numbers over time (Figure 1C and Supplemental Figure 1C). These results suggested that mDia1 depletion did not cause overall viability defects allowing us to study the role of mDia1 in leukemia cell migration *in vitro* and progression *in vivo* without the potential confounding factor of reduced viability due to impaired mDia1 expression.

### mDia1-deficient B-ALL cells have impaired ability to undergo transendothelial migration

**Figure 2:**
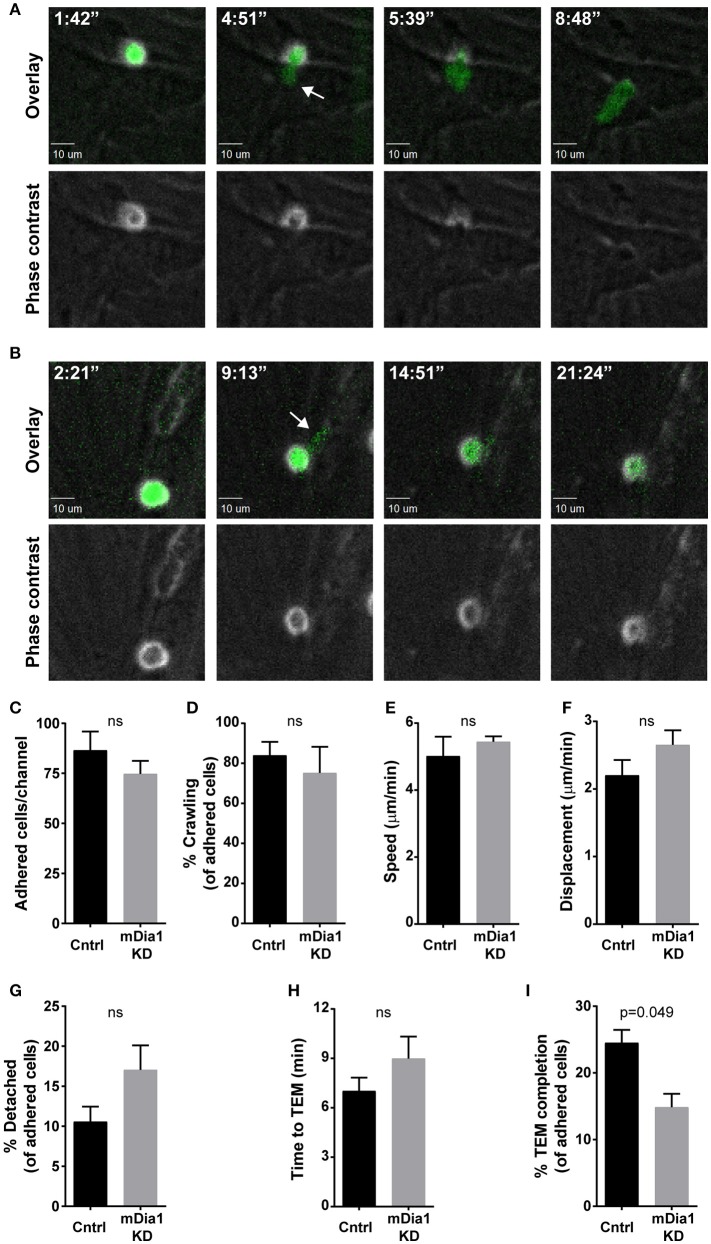
mDia1 deficiency impairs transendothelial migration of B-ALL cells. Fluorescently-labeled control and mDia1 KD B-ALL cells were introduced into flow chambers with a monolayer of bEnd.3 endothelial cells in the presence of CXCL12 and then maintained under a shear flow of 2 dyne/cm^2^ and imaged for 30 min using a spinning-disk confocal microscope. Phase contrast and fluorescence images were captured during time-lapse imaging every 15–25 s as indicated. **(A)** Representative images of a control B-ALL cell undergoing transendothelial migration (TEM). Top panels, overlay of ZsGreen fluorescence and phase contrast images; bottom panels, phase contrast images. As the control cell completes transendothelial migration the light ring around the cell body in the Phase contrast channel (bottom panels) disappears step-wise. The white arrow indicates a cellular protrusion initiating the transendothelial migration process. Time is in min:sec. **(B)** Representative images of an mDia1 KD B-ALL cell attempting and failing to complete transendothelial migration (depicted as in **A**). **C**. Similar adhesion of control and mDia1 KD B-ALL cells to the endothelial monolayer. (**D–F)** Control and mDia1 KD cells have equivalent crawling behavior on the endothelial cell monolayer. (**G**) mDia1 depletion does not significantly alter B-ALL detachment from the endothelial monolayer. **(H)** mDia1 KD cells take a similar amount of time to initiate TEM. (**I)** mDia1 deficiency significantly reduces the capacity of B-ALL cells to complete transendothelial migration. Data in **(A,B)** are representative of 4 independent experiments; data in **(C–I)** are the average of 4 independent experiments, with at least 61 cells/group for each experiment **(C,D,G–I)** or at least 27 cells/group for each experiment **(E,F)**. Error bars are the SEM.

Given the importance of cell migration for leukemia dissemination, and the reported role of mDia1 in T cell migration (20, 21, 24) and of the related Formin FMNL1 in leukemia migration (27), we investigated if mDia1 depletion affected the ability of B-ALL cells to complete the various steps of transendothelial migration. For these experiments we set up an *in vitro* reductionist system to visualize and analyze the process of transendothelial migration in real-time by time-lapse microscopy. Transendothelial migration is a multi-step process that entails the capture of leukocytes on the endothelial monolayer by an initial rolling step, followed by firm adhesion to resist vascular shear forces (4). Subsequently, leukocytes can migrate over the endothelial monolayer and finally migrate through the endothelial barrier to complete the extravasation process. With our imaging system we analyzed these transendothelial migration steps on endothelial cells under physiological shear flow (Figures 2A,B). Using phase contrast imaging, leukocytes above the endothelial monolayer display a white phase contrast halo that disappears step-wise as the leukocyte undergoes transendothelial migration (Figure 2A, bottom panels, and Supplemental Video 1). Comparing control and mDia1 KD cells we found no significant difference in B-ALL cell adhesion to the endothelial monolayer (Figure 2C). The fraction of leukemia cells crawling on the endothelial monolayer and their motility characteristics were also not altered in mDia1 KD B-ALL cells (Figures 2D–F). Furthermore, detachment under flow (Figure 2G) and the time to initiate transendothelial migration (Figure 2H) were not significantly different in mDia1 KD cells. However, we identified a significant impairment of the adhered mDia1-deficient B-ALL cells in completing the process of transendothelial migration compared to control B-ALL cells (Figure 2I and Supplemental Video 2).

### Depletion of mDia1 reduces leukemia cell chemotaxis

**Figure 3:**
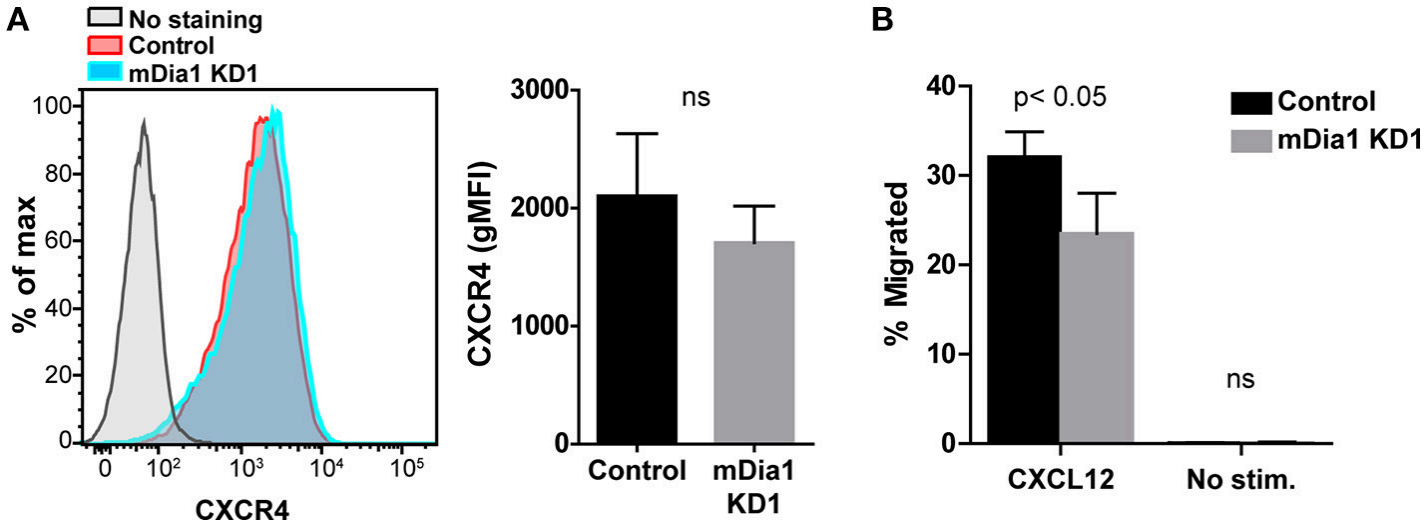
mDia1 depletion reduces the ability of B-ALL cells to undergo chemotaxis. **(A)** Left, representative flow cytometry staining for CXCR4 of control and mDia1 KD B-ALL cells. Right, quantification of CXCR4 surface expression on control and mDia1 KD cells. **(B)** Quantification of the percentage of chemotactic migration with or without CXCL12 through 5 μm pore transwell membranes of control and mDia1 KD B-ALL cells. Data in **(A)** are from 4 independent experiments; data in **(B)** are the average of 5 independent experiments. Error bars are the SEM.

Chemokines have been shown to affect various steps of the transendothelial migration process (4, 35). Having seen reduced transendothelial migration of mDia1 KD B-ALL cells, as a potential mechanism for this reduced migration, we subsequently analyzed if leukemia cells lacking mDia1 would be affected in their capacity to respond to chemokine stimulation. The CXCL12-CXCR4 axis plays an important role for homing and engraftment of leukemia cells to the bone marrow and other tissues (36–40). We therefore first analyzed the expression levels of the CXCR4 receptor on control and mDia1 KD B-ALL cells and found no significant difference in CXCR4 expression (Figure 3A and Supplemental Figure 2A). Next, using the transwell chamber system, we determined if the ability to migrate in response to CXCL12 was affected by mDia1 depletion in B-ALL cells. Our data showed that B-ALL cells migrated in response to CXCL12 stimulation, and that migration through 5 μm pore transwell membranes in response to a CXCL12 gradient was significantly impaired in both mDia1 KD B-ALL cell lines (Figure 3B and Supplemental Figure 2B).

### mDia1 promotes leukemia engraftment

**Figure 4:**
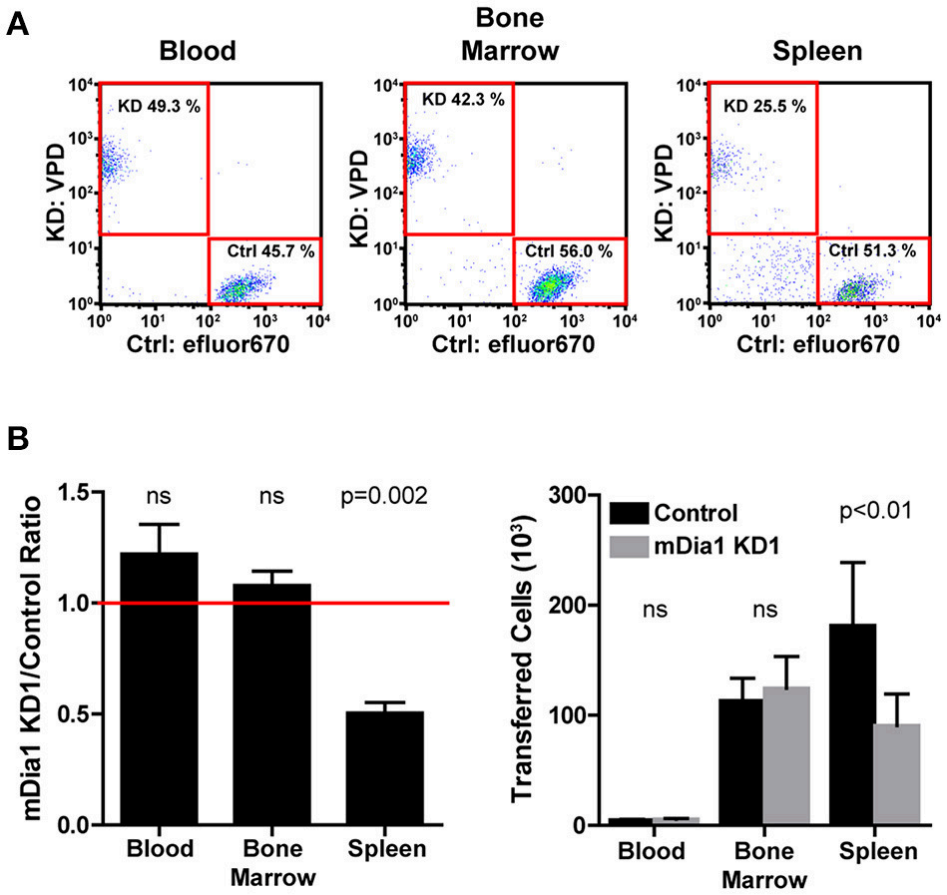
Depletion of mDia1 decreases engraftment of B-ALL cells. Differentially dye-labeled control and mDia1 KD B-ALL cells were intra-venously co-transferred at a 1:1 ratio in CD45.1+ recipient mice. The number of B-ALL cells in the blood, bone marrow, and spleen of recipient mice was determined 24 h post-transfer by flow cytometry. **(A)** Representative flow cytometry plots of differentially-labeled control and mDia1 KD cells recovered from the indicated tissues. The transferred B-ALL cells were identified by gating on CD45.1-negative/ZsGreen-positive cells (not shown). **(B)** Quantification of the ratio and number of control and mDia1 KD B-ALL cells in the indicated tissues. A ratio below 1.0, indicated by the horizontal red line, shows reduced numbers of the mDia1 KD B-ALL cells. Data in **(A)** are representative of 4 experiments; data in **(B)** are the average of 4 experiments each with 2 mice/group. Error bars are the SEM.

The defects in chemotaxis toward CXCL12 and in transendothelial migration suggested that mDia1 could have a role in regulating the ability of leukemia cells to migrate and engraft *in vivo*. Therefore, using an adoptive transfer model, we measured the engraftment capacity of control and mDia1 KD B-ALL cells. Using short-term transfer assays, we analyzed the number of B-ALL cells in the blood, bone marrow and spleen of recipient mice by flow cytometry 24 h after intravenous adoptive transfer. To minimize variability due to the transfer procedure or the recipient mice, we co-transferred equal numbers of differentially-fluorescently labeled control and mDia1 KD cells. We used recipient mice expressing the congenic marker CD45.1 to readily distinguish endogenous cells from the transferred B-ALL which are CD45.2^+^. Using this experimental system, our data showed that mDia1 KD B-ALL cells had a significant reduction in their ability to engraft and colonize the spleen 24 h after transfer (Figures 4A,B). We further validated that this engraftment defect was specific to mDia1 depletion by analyzing the engraftment capacity of B-ALL cells transduced with the second mDia1 shRNA. Our data confirmed that mDia1 deficient B-ALL cells are significantly impaired in colonizing the spleen (Supplemental Figure 3).

We then analyzed the surface expression of the integrins LFA-1 (αLβ2, CD11a/CD18) and VLA-4 (α4β1, CD49d/CD29), adhesion proteins that can play a homing role during trafficking (4). We found comparable CD11a expression between control and mDia1 KD cells (Supplemental Figures 4A,C). In our analysis of CD49d, we detected differences in CD49d expression that were not consistent between the two different mDia1 KD cells transduced with either mDia1 shRNA (Supplemental Figures 4B,D), suggesting that these differences are not responsible for the impairment of mDia1 KD cell migration and engraftment seen consistently with both mDia1 KD cells.

### mDia1 deficiency reduces leukemia progression *in vivo*

**Figure 5:**
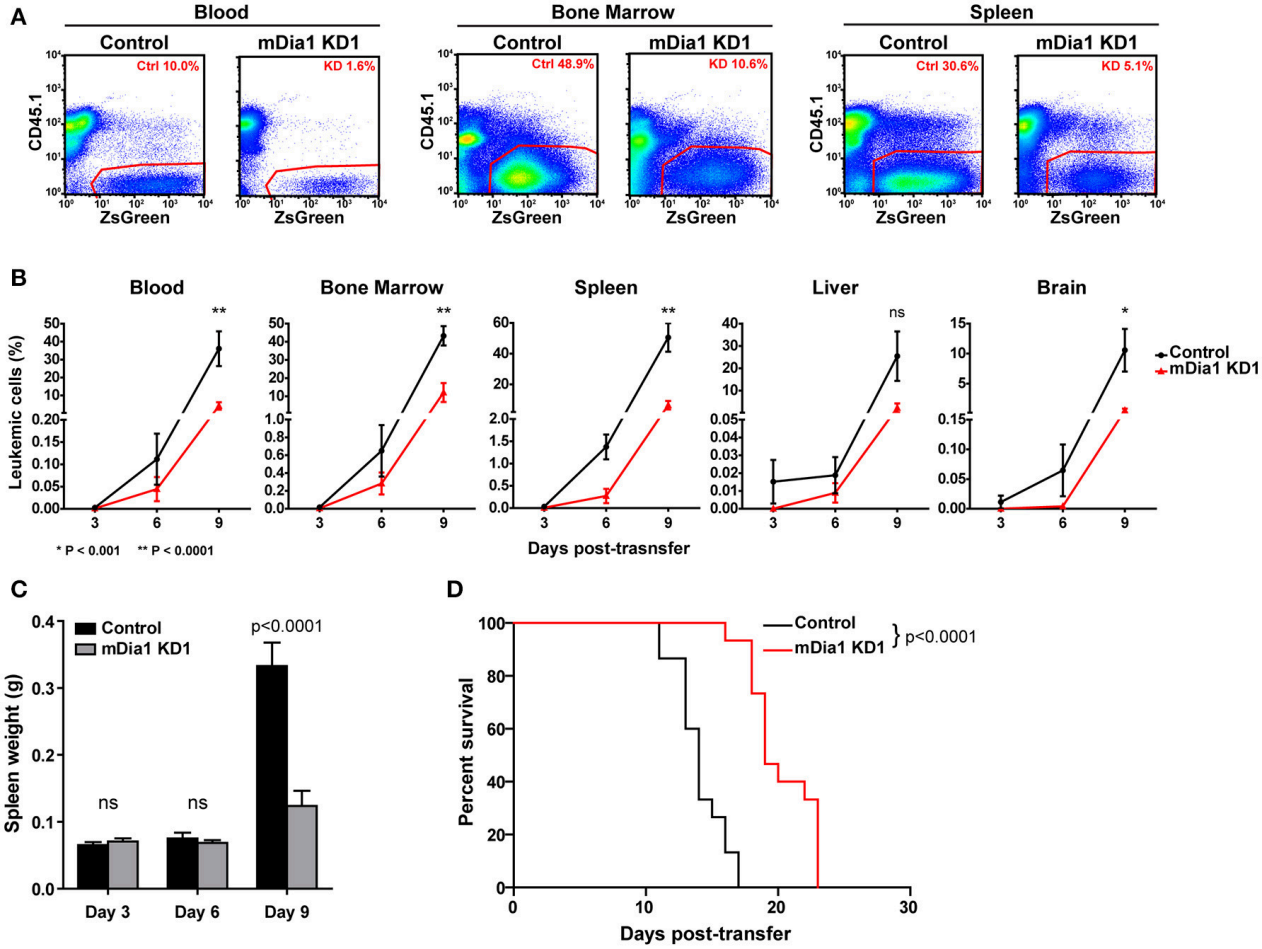
mDia1 deficiency reduces leukemia progression *in vivo* and prolongs survival. Control or mDia1 KD B-ALL cells were transferred intra-venously into CD45.1+ recipient mice. **(A,B)** Every 3 days, the number of transferred B-ALL cells was quantified by flow cytometry in randomly selected pairs of recipient mice (1 control and 1 mDia1 KD). The transferred B-ALL cells were identified by gating on CD45.1-negative/ZsGreen-positive cells. **(A)** Representative flow cytometry plots of control and mDia1 KD B-ALL cells recovered from recipient mice in the indicated tissues. **(B)** Quantification of the frequency of control and mDia1 KD cells in the indicated tissues over time. **(C)** Reduced spleen colonization by mDia1-deficient B-ALL cells. As a readout of spleen colonization by the leukemia, the weight of the spleen in randomly selected pairs of recipient mice was determined every 3 days. **(D)** mDia1 depletion in leukemia cells prolongs survival. Using the above experimental set up, the recipient mice were monitored daily for signs of leukemia and euthanized once signs of morbidity were detected. Data in **(A)** are representative of 3 experiments; data in **(B,C)** are the average of 3 experiments each with 2 mice/group/time-point; data in **(D)** are pooled from 3 independent experiments each with cohorts of 5 mice/group/experiment. Error bars are the SEM.

We next analyzed the *in vivo* progression and dissemination of control and mDia1-deficient leukemia cells over time. For these experiments, we transferred control or mDia1 KD cells in separate recipient mice since fluorescent dye labels would dilute too much over the course of the experiment to reliably identify the control and KD populations. Furthermore, this experimental setup would also avoid any confounding effects due to the presence of control B-ALL cells helping the mDia1 KD B-ALL invade and colonize various tissues. For these experiments, every 3 days, we determined the number of CD45.2^+^ control or mDia1 KD B-ALL cells in various tissues of CD45.1^+^ recipient mice (Figures 5A,B). Our analysis determined that, over the course of leukemia progression, mDia1-deficient B-ALL cells have a significant reduction in their dissemination and expansion in various tissues including: Blood, Bone Marrow, Spleen, and Brain (Figure 5B).

In this leukemia model the spleen is a main site of leukemia colonization and pathology, therefore, in the above experiment we also measured the leukemia burden in the spleen of recipient mice by determining the weights of the spleens of recipient mice during this time-course experiment. Our data showed significantly smaller spleens in the mice receiving mDia1-deficient B-ALL cells at the end of the time-course (Figure 5C, day 9 time-point).

### mDia1 depletion in leukemia cells prolongs survival

Finally, we investigated if the defects seen in spleen engraftment and reduced leukemic cells in tissues during leukemia progression of mDia1 KD B-ALL cells would result in improved survival of the recipient mice. To this end, we analyzed the survival of recipient mice receiving control or mDia1 KD B-ALL cells. For these experiments, we transferred control or mDia1 KD B-ALL cells into wild-type immunocompetent recipient mice and then determined leukemia incidence over time. Our data showed a significant extension of the survival of recipient mice transferred with mDia1 KD cells compared to control B-ALL cells (Figure 5D and Supplemental Figure 5). Overall, our data suggest that mDia1 regulates the ability of leukemia cells to extravasate and engraft into the spleen promoting leukemia progression.

## Discussion

Here we report that the Formin mDia1 promotes leukemia migration and progression *in vivo*. Although some Formins have been shown to affect cell proliferation (27), our data suggests that the effect of mDia1 deficiency on leukemia progression *in vivo* is more related to the ability of the leukemia cells to engraft and disseminate rather than effects on their proliferation capacity or viability.

Formins, and mDia1 in particular, can modulate many cellular processes including cell polarity and migration of both normal and transformed cells by regulating microtubules and actin networks (14–16). mDia1 has been shown to mediate actin polymerization in response to chemokine and antigen stimulation in lymphocytes (20, 21), and to regulate leukocyte motility (20, 21, 23, 41). Consistent with these findings, we demonstrated that mDia1-deficient B-ALL cells have impaired chemotaxis and have reduced capacity to complete transendothelial migration through endothelial cell barriers. Interestingly, a previous report found that B cells lacking mDia1 were able to undergo *in vitro* chemotaxis normally (20). On the other hand, our data shows that mDia1-deficient transformed pre-B cells are clearly impaired in chemotaxis and transendothelial migration. Furthermore, our results also indicate that the migratory defect of mDia1-deficient B-ALL cells causes reduced engraftment and dissemination of leukemia cells into tissues *in vivo*.

mDia1 can localize to the tips of filopodia (42, 43, 44), which are elongated membrane protrusions containing parallel bundles of linear actin filaments. Filopodia have been suggested to serve as environmental sensors and possibly guide migration (45). Therefore, consistent with our data, a mechanism by which cells deficient in mDia1 have impaired migratory activity and reduced transendothelial migration could be caused by reduced formation and function of filopodia in response to chemokine stimulation. Additionally, mDia1 has been implicated in cross-talk between the actin and microtubule cytoskeletons (46–48). Thus, a further mechanism by which mDia1 deficiency could impair leukemia cell migration is by disrupting coordination of the actin and microtubule cytoskeletons during transendothelial migration.

Previous studies in tumors of non-lymphoid origin have suggested a role for mDia1 in promoting cancer invasion, migration and consequently metastasis (48–50). These previous studies have focused on the role of mDia1 in mediating morphological changes that enable malignant cells to migrate out of their native tissue environment. Our finding that mDia1 promotes transendothelial migration of leukemia cells may have additional implications for the dissemination of other cancer types once they enter the blood stream, suggesting that mDia1 also plays an important role in extravasation and tissue infiltration. Interestingly, mDia1 has been shown to be highly expressed in activated lymphocytes, including transformed lymphocytes (24), which could have implications on the ability of these cells to disseminate.

Overall, our findings show that mDia1 is a positive regulator of leukemia progression by promoting leukemia cell transendothelial migration and engraftment, thereby contributing to leukemia progression *in vivo*. Our data showing prolonged survival of recipient mice receiving mDia1-deficient leukemia cells suggest that this Formin, and the signaling pathways that regulate its activity, can be potential therapeutic targets for the treatment of ALL by preventing leukemia cells from reaching and colonizing niches that enable tumor progression. However, the relatively widespread tissue expression of mDia1 and the current lack of a selective inhibitor of mDia1 may pose a challenge for therapeutic targeting of this Formin protein.

## Author contributions

ST performed the majority of the experiments and participated in designing some experiments; EW and SK performed the *in vitro* transendothelial migration experiments. EW, JC and RL participated in performing some of the *in vivo* disease progression experiments. ST and JJ analyzed and interpreted the data, made figures, and wrote the manuscript; JJ conceived and designed the experiments, and supervised the overall project.

### Conflict of interest statement

The authors declare that the research was conducted in the absence of any commercial or financial relationships that could be construed as a potential conflict of interest.

## References

[1] Yeoh EJ Ross ME Shurtleff SA Williams WK Patel D Mahfouz R. Classification, subtype discovery, and prediction of outcome in pediatric acute lymphoblastic leukemia by gene expression profiling. Cancer Cell (2002) 1:133–43. 10.1016/S1535-6108(02)00032-6 12086872

[2] Pals ST de Gorter DJ Spaargaren M. Lymphoma dissemination: the other face of lymphocyte homing. Blood (2007) 110:3102–11. 10.1182/blood-2007-05-075176 17656647

[3] Reuss-Borst MA Klein G Waller HD Muller CA. Differential expression of adhesion molecules in acute leukemia. Leukemia (1995) 9:869–74. 7539515

[4] Ley K Laudanna C Cybulsky MI Nourshargh S. Getting to the site of inflammation: the leukocyte adhesion cascade updated. Nat Rev Immunol. (2007) 7:678–89. 10.1038/nri2156 17717539

[5] Butcher EC. Leukocyte-endothelial cell recognition: three (or more) steps to specificity and diversity. Cell (1991) 67:1033–6. 10.1016/0092-8674(91)90279-8 1760836

[6] Nourshargh S Hordijk PL Sixt M. Breaching multiple barriers: leukocyte motility through venular walls and the interstitium. Nat Rev Molecul Cell Biol. (2010) 11:366–78. 10.1038/nrm2889 20414258

[7] Lammermann T Sixt M. Mechanical modes of 'amoeboid' cell migration. Curr. Opin. Cell Biol. (2009) 21:636–44. 10.1016/j.ceb.2009.05.003 19523798

[8] Krummel MF Friedman RS Jacobelli J Modes and mechanisms of T cell motility: roles for confinement and Myosin-IIA. Curr. Opin. Cell Biol. (2014) 30C:9–16. 10.1016/j.ceb.2014.05.003 PMC4178009 24905977

[9] Ridley AJ. Life at the leading edge. Cell (2011) 145:1012–22. 10.1016/j.cell.2011.06.010 21703446

[10] Meacham CE Ho EE Dubrovsky E Gertler FB Hemann MT. *In vivo* RNAi screening identifies regulators of actin dynamics as key determinants of lymphoma progression. Nat. Genet. (2009) 41:1133–7 10.1038/ng.451 19783987 PMC2756700

[11] Wigton EJ Thompson SB Long RA Jacobelli J. Myosin-IIA regulates leukemia engraftment and brain infiltration in a mouse model of acute lymphoblastic leukemia. J. Leukocyte Biol. (2016) 100:143–153. 10.1189/jlb.1A0815-342R 26792819 PMC5627497

[12] Heng TS Painter MW. The immunological genome project: networks of gene expression in immune cells. Nat. Immunol. (2008) 9:1091–4. 10.1038/ni1008-1091 18800157

[13] Huse M Lillemeier BF Kuhns MS Chen DS Davis MM. T cells use two directionally distinct pathways for cytokine secretion. Nat. Immunol. (2006) 7:247–55. 10.1038/ni1304 16444260

[14] Faix J Grosse R. Staying in shape with formins. Dev. Cell (2006) 10:693–706. 10.1016/j.devcel.2006.05.001 16740473

[15] Breitsprecher D Goode BL. Formins at a glance. J Cell Sci. (2013) 126(Pt 1):1–7. 10.1242/jcs.107250 23516326 PMC3603506

[16] Chesarone MA DuPage AG Goode BL. Unleashing formins to remodel the actin and microtubule cytoskeletons. Nat Rev Molecul Cell Biol. (2010) 11:62–74. 10.1038/nrm2816 19997130

[17] Lizarraga F Poincloux R Romao M Montagnac G Le Dez G Bonne I. Diaphanous-related formins are required for invadopodia formation and invasion of breast tumor cells. Cancer Res. (2009) 69:2792–800. 10.1158/0008-5472.CAN-08-3709 19276357

[18] Sarmiento C Wang W Dovas A Yamaguchi H Sidani M El-Sibai M. WASP family members and formin proteins coordinate regulation of cell protrusions in carcinoma cells. J Cell Biol. (2008) 180:1245–60. 10.1083/jcb.200708123 18362183 PMC2290849

[19] Kitzing TM Sahadevan AS Brandt DT Knieling H Hannemann S Fackler OT. Positive feedback between Dia1, LARG, and RhoA regulates cell morphology and invasion. Genes Dev. (2007) 21:1478–83. 10.1101/gad.424807 17575049 PMC1891425

[20] Sakata D Taniguchi H Yasuda S Adachi-Morishima A Hamazaki Y Nakayama R. Impaired T lymphocyte trafficking in mice deficient in an actin-nucleating protein, mDia1. J Exp Med. (2007) 204:2031–8. 10.1084/jem.20062647 17682067 PMC2118705

[21] Eisenmann KM West RA Hildebrand D Kitchen SM Peng J Sigler R. T cell responses in mammalian diaphanous-related formin mDia1 knock-out mice. J Biol Chem. (2007) 282:25152–8. 10.1074/jbc.M703243200 17595162

[22] Yayoshi-Yamamoto S Taniuchi I Watanabe T. FRL, a novel formin-related protein, binds to Rac and regulates cell motility and survival of macrophages. Mol Cell Biol. (2000) 20:6872–81. 10.1128/MCB.20.18.6872-6881.2000 10958683 PMC86228

[23] Tanizaki H Egawa G Inaba K Honda T Nakajima S Moniaga CS. Rho-mDia1 pathway is required for adhesion, migration, and T-cell stimulation in dendritic cells. Blood (2010) 116:5875–84. 10.1182/blood-2010-01-264150 20881208

[24] Vicente-Manzanares M Rey M Perez-Martinez M Yanez-Mo M Sancho D Cabrero JR. The RhoA effector mDia is induced during T cell activation and regulates actin polymerization and cell migration in T lymphocytes. J Immunol. (2003) 171:1023–34. 10.4049/jimmunol.171.2.1023 12847276

[25] Favaro PM Traina F Vassallo J Brousset P Delsol G Costa FF. High expression of FMNL1 protein in T non-Hodgkin's lymphomas. Leukemia Res. (2006) 30:735–8. 10.1016/j.leukres.2005.10.003 16494944

[26] Favaro PM de Souza Medina S Traina F Basseres DS Costa FF Saad ST. Human leukocyte formin: a novel protein expressed in lymphoid malignancies and associated with Akt. Biochem Biophys Res Commun. (2003) 311:365–71. 10.1016/j.bbrc.2003.10.012 14592423

[27] Favaro P Traina F Machado-Neto JA Lazarini M Lopes MR Pereira JK. FMNL1 promotes proliferation and migration of leukemia cells. J Leukoc Biol. (2013) 94:503–12. 10.1189/jlb.0113057 23801653

[28] Williams RT Roussel MF Sherr CJ. Arf gene loss enhances oncogenicity and limits imatinib response in mouse models of Bcr-Abl-induced acute lymphoblastic leukemia. Proc Natl Acad Sci USA. (2006) 103:6688–93. 10.1073/pnas.0602030103 16618932 PMC1440588

[29] Estin ML Thompson SB Traxinger B Fisher MH Friedman RS Jacobelli J. Ena/VASP proteins regulate activated T-cell trafficking by promoting diapedesis during transendothelial migration. Proc Natl Acad Sci USA. (2017) 114:E2901–10. 10.1073/pnas.1701886114 28320969 PMC5389297

[30] Williams RT den Besten W Sherr CJ. Cytokine-dependent imatinib resistance in mouse BCR-ABL+, Arf-null lymphoblastic leukemia. Genes Dev. (2007) 21:2283–7. 10.1101/gad.1588607 17761812 PMC1973142

[31] Boulos N Mulder HL Calabrese CR Morrison JB Rehg JE Relling MV. Chemotherapeutic agents circumvent emergence of dasatinib-resistant BCR-ABL kinase mutations in a precise mouse model of Philadelphia chromosome-positive acute lymphoblastic leukemia. Blood (2011) 117:3585–95. 10.1182/blood-2010-08-301267 21263154 PMC3072880

[32] Bohnert KA Willet AH Kovar DR Gould KL. Formin-based control of the actin cytoskeleton during cytokinesis. Biochem Soc Trans. (2013) 41:1750–4. 10.1042/BST20130208 24256286 PMC5563852

[33] Castrillon DH W asserman SA. Diaphanous is required for cytokinesis in Drosophila and shares domains of similarity with the products of the limb deformity gene. Development (1994) 120:3367–77. 7821209 10.1242/dev.120.12.3367

[34] Kato T Watanabe N Morishima Y Fujita A Ishizaki T Narumiya S. Localization of a mammalian homolog of diaphanous, mDia1, to the mitotic spindle in HeLa cells. J Cell Sci. (2001) 114(Pt 4):775–84. 11171383 10.1242/jcs.114.4.775

[35] Alon R Shulman Z. Chemokine triggered integrin activation and actin remodeling events guiding lymphocyte migration across vascular barriers. Exp Cell Res. (2011) 317:632–41. 10.1016/j.yexcr.2010.12.007 21376176

[36] Sipkins DA Wei X Wu JW Runnels JM Cote D Means TK. *in vivo* imaging of specialized bone marrow endothelial microdomains for tumour engraftment. Nature (2005) 435:969–73. 10.1038/nature03703 15959517 PMC2570168

[37] Spiegel A Kollet O Peled A Abel L Nagler A Bielorai B. Unique SDF-1-induced activation of human precursor-B ALL cells as a result of altered CXCR4 expression and signaling. Blood (2004) 103:2900–7. 10.1182/blood-2003-06-1891 15070661

[38] Shen W Bendall LJ Gottlieb DJ Bradstock KF. The chemokine receptor CXCR4 enhances integrin-mediated *in vitro* adhesion and facilitates engraftment of leukemic precursor-B cells in the bone marrow. Exp Hematol. (2001) 29:1439–47. 10.1016/S0301-472X(01)00741-X 11750103

[39] Burger JA Peled A. CXCR4 antagonists: targeting the microenvironment in leukemia and other cancers. Leukemia (2009) 23:43–52. 10.1038/leu.2008.299 18987663

[40] Tavor S Petit I. Can inhibition of the SDF-1/CXCR4 axis eradicate acute leukemia? Semin Cancer Biol. (2010) 20:178–85. 10.1016/j.semcancer.2010.07.001 20637871

[41] Vargas P Maiuri P Bretou M Saez PJ Pierobon P Maurin M Innate control of actin nucleation determines two distinct migration behaviours in dendritic cells. Nat Cell Biol. (2016) 18:43–53. 10.1038/ncb3284 26641718 PMC5885286

[42] Naj X Hoffmann AK Himmel M Linder S. The formins FMNL1 and mDia1 regulate coiling phagocytosis of Borrelia burgdorferi by primary human macrophages. Infect Immun. (2013) 81:1683–95. 10.1128/IAI.01411-12 23460512 PMC3647995

[43] Goh WI Sudhaharan T Lim KB Sem KP Lau CL Ahmed S. Rif-mDia1 interaction is involved in filopodium formation independent of Cdc42 and Rac effectors. J Biol Chem. (2011) 286:13681–94. 10.1074/jbc.M110.182683 21339294 PMC3075712

[44] Goh WI Lim KB Sudhaharan T Sem KP Bu W Chou AM. mDia1 and WAVE2 proteins interact directly with IRSp53 in filopodia and are involved in filopodium formation. J Biol Chem. (2012) 287:4702–14. 10.1074/jbc.M111.305102 22179776 PMC3281667

[45] Mellor H. The role of formins in filopodia formation. Biochim Biophys Acta (2010) 1803:191–200. 10.1016/j.bbamcr.2008.12.018 19171166

[46] Zaoui K Honore S Isnardon D Braguer D Badache A. Memo-RhoA-mDia1 signaling controls microtubules, the actin network, and adhesion site formation in migrating cells. J Cell Biol. (2008) 183:401–8. 10.1083/jcb.200805107 18955552 PMC2575782

[47] Wen Y Eng CH Schmoranzer J Cabrera-Poch N Morris EJ Chen M. EB1 and APC bind to mDia to stabilize microtubules downstream of Rho and promote cell migration. Nat Cell Biol. (2004) 6:820–30. 10.1038/ncb1160 15311282

[48] Yamana N Arakawa Y Nishino T Kurokawa K Tanji M Itoh RE. The Rho-mDia1 pathway regulates cell polarity and focal adhesion turnover in migrating cells through mobilizing Apc and c-Src. Mol Cell Biol. (2006) 26:6844–58. 10.1128/MCB.00283-06 16943426 PMC1592856

[49] Lin YN Izbicki JR Konig A Habermann JK Blechner C Lange T. Expression of DIAPH1 is up-regulated in colorectal cancer and its down-regulation strongly reduces the metastatic capacity of colon carcinoma cells. Int J Cancer (2014) 134:1571–82. 10.1002/ijc.28486 24105619

[50] Kim D Jung J You E Ko P Oh S Rhee S. mDia1 regulates breast cancer invasion by controlling membrane type 1-matrix metalloproteinase localization. Oncotarget (2016) 7:17829–43. 10.18632/oncotarget.7429 26893363 PMC4951253

