# Supplementary material for: The Formin mDia1 Regulates Acute Lymphoblastic Leukemia Engraftment, Migration, and Progression in vivo
Source: Front Oncol. 2018 Sep 20;8:389. doi: 10.3389/fonc.2018.00389 (PMC6158313; doi:10.3389/fonc.2018.00389)
Supplement: Supplementary file 3 [file Data_Sheet_2.PDF]

Supplementary Material

The Formin mDia1 regulates acute lymphoblastic leukemia engraftment, migration, and progression in vivo

Scott B. Thompson, Eric J. Wigton, S. Harsha Krovi, Jeffrey W. Chung, Robert A. Long, and Jordan Jacobelli*

*** Correspondence:** Corresponding author: jacobellij@njhealth.org

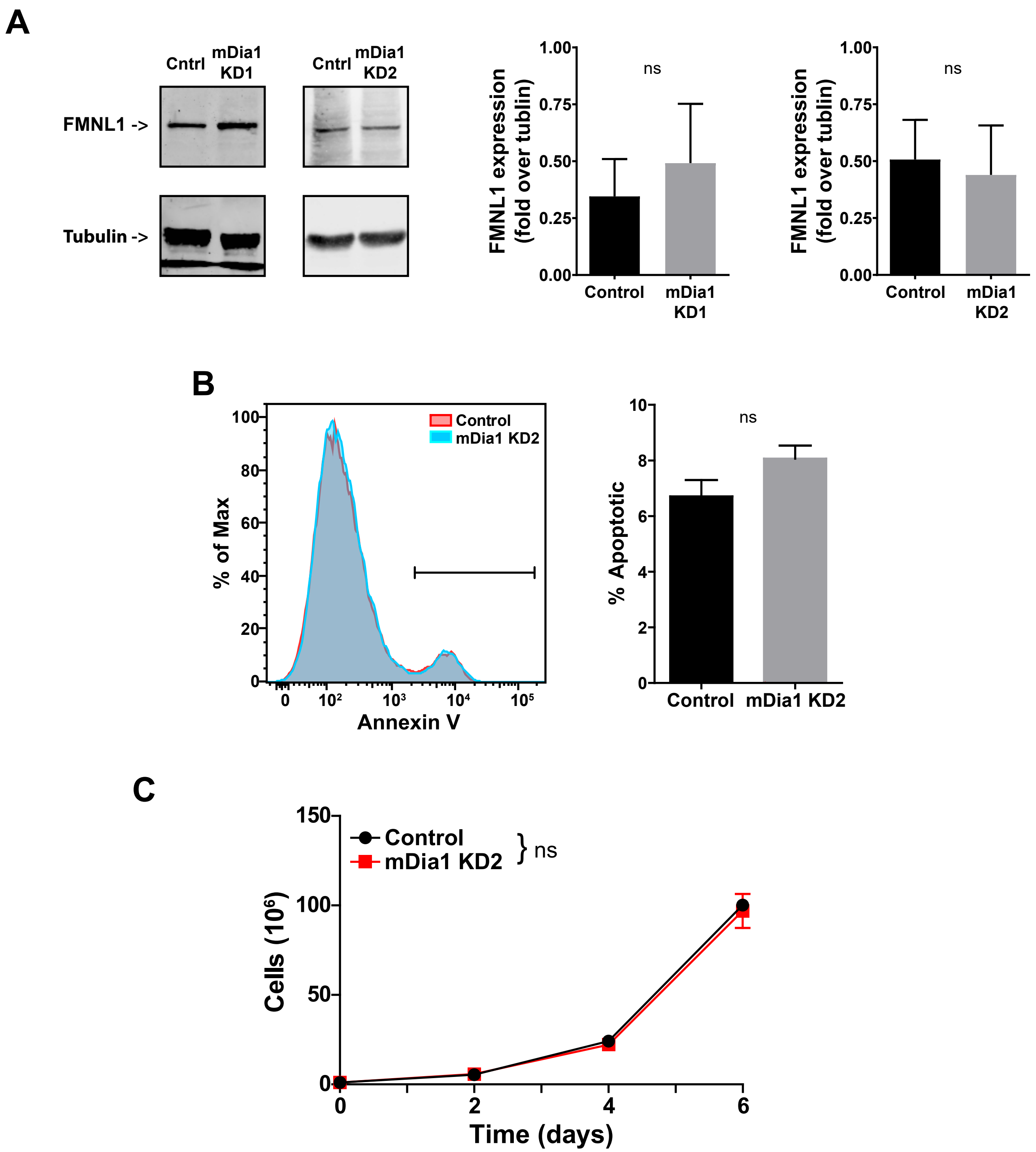

Supplementary Figure 1. mDia1 knock-down does not affect expression of FMNL1 and does not alter B-ALL cell viability or proliferation. A. Expression of FMNL1, the other main Formin expressed in lymphocytes, was analyzed in control and mDia1 knock-down (KD) B-ALL cells. Left panels, Western blot analysis of cell lysates from control and mDia1 KD cells. Tubulin staining is shown for normalization purposes. Right panels, quantification of FMNL1 expression relative to tubulin. **B-C.** B-ALL cells were transduced with a second shRNA construct targeting mDia1 (mDia1 KD2) or with a control shRNA. **B**. B-ALL cell apoptosis is not increased in mDia1 KD cells. Left, representative flow cytometry staining for Annexin V of control and mDia1 KD cells. Right, quantification of the frequency of apoptotic cells. **C**. mDia1 KD does not impair B-ALL proliferation. In vitro proliferation of B-ALL cells over the course of 6 days. Data in A are from 2 independent experiments, data in B and C are the average of at least 3 independent experiments. Error bars are the SEM.

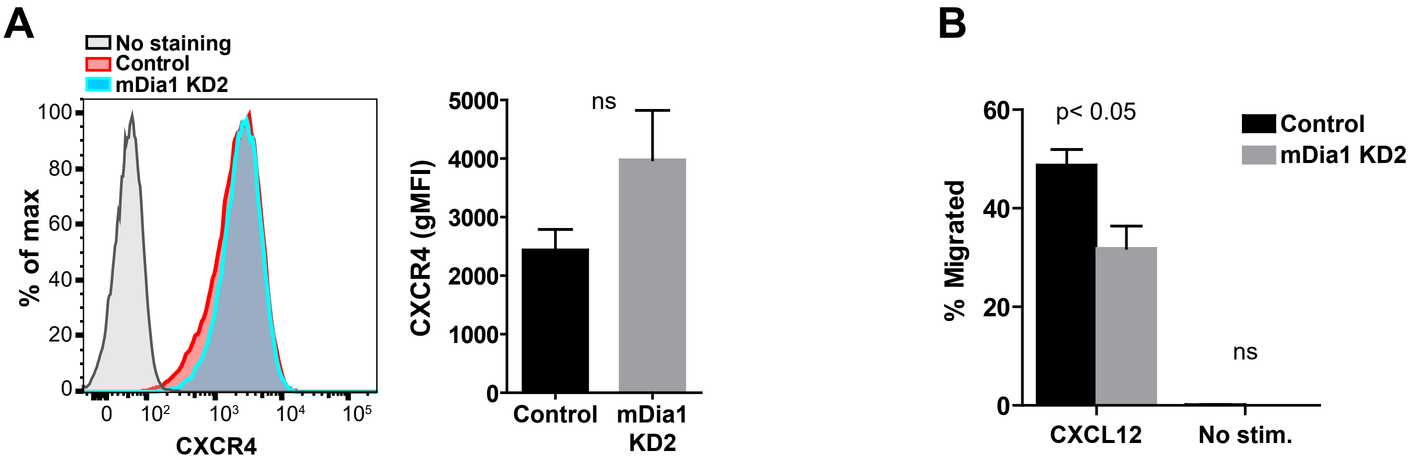

Supplementary Figure 2. mDia1 depletion reduces the ability of B-ALL cells to undergo chemotaxis. B-ALL cells were transduced with a second shRNA construct targeting mDia1 (mDia1 KD2) or with a control shRNA. **A**. Left, representative flow cytometry staining for CXCR4 of control and mDia1 KD cells. Right, quantification of CXCR4 surface expression on control and mDia1 KD cells. **B**. Quantification of the percentage of chemotactic migration with or without CXCL12 trough 5µm pore transwell membranes of control and mDia1 KD B-ALL cells. Data in A-C are from 4 independent experiments; data in D are the average of 3 independent experiments. Error bars are the SEM.

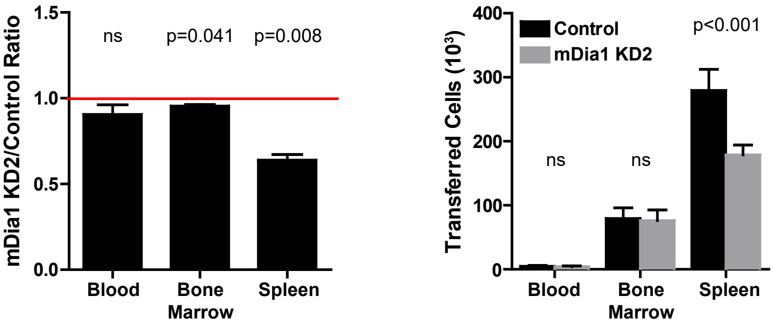

Supplementary Figure 3. Depletion of mDia1 decreases engraftment of B-ALL cells. B-ALL cells were transduced with a second shRNA construct targeting mDia1 (mDia1 KD2) or with a control shRNA. Differentially dye-labeled control and mDia1 KD were then intra-venously co-transferred at a 1:1 ratio in CD45.1+ recipient mice. The number of B-ALL cells in the blood, bone marrow and spleen of recipient mice was determined 24 hours post-transfer by flow cytometry. Quantification of the ratio (left panel) and number (right panel) of control and mDia1 KD cells in the indicated tissues. A ratio below 1.0, indicated by the horizontal red line, shows reduced numbers of the mDia1 KD B-ALL cells. Data are the average of 3 experiments each with 2 mice/group. Error bars are the SEM.

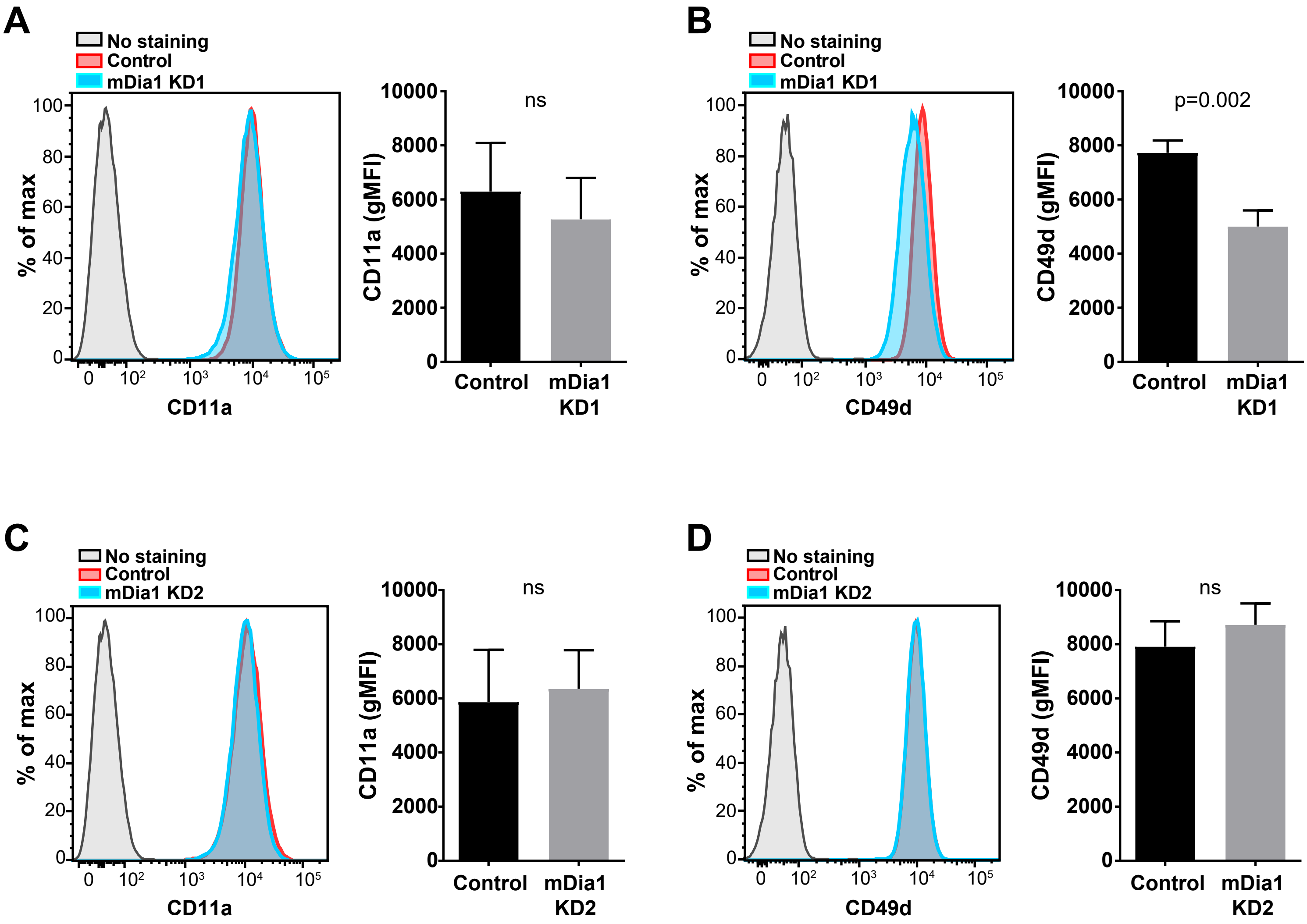

Supplementary Figure 4. Expression of CD11a and CD49d integrins on mDia1-deficient B-ALL cells. B-ALL cells were transduced with shRNA constructs targeting mDia1 or with a control shRNA and stained for integrin expression. **A** and **C**. Left, representative flow cytometry staining for CD11a of control and mDia1 KD cells. Right, quantification of CD11a surface expression on control and mDia1 KD cells. **B** and **D**. Left, representative flow cytometry staining for CD49d of control and mDia1 KD cells. Right, quantification of CD49d surface expression on control and mDia1 KD cells. Data are from 4 independent experiments. Error bars are the SEM.

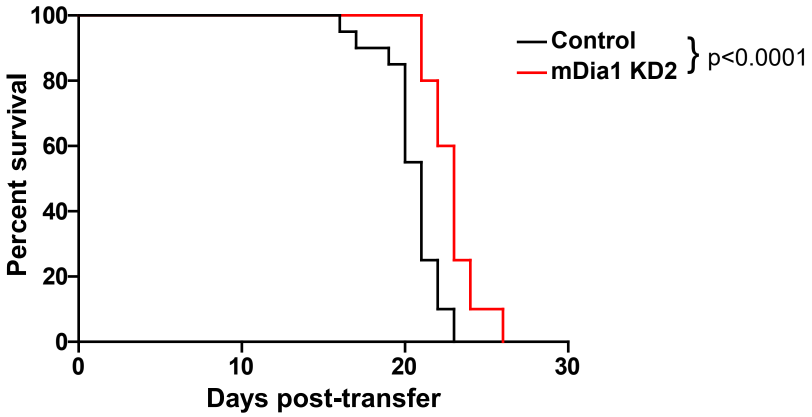

Supplementary Figure 5. mDia1 deficiency in leukemia cells prolongs survival. B-ALL cells were transduced with a second shRNA construct targeting mDia1 (mDia1 KD2) or with a control shRNA. Control or mDia1 KD B-ALL cells were then transferred intra-venously into CD45.1+ recipient mice. The recipient mice were monitored daily for signs of leukemia and euthanized once signs of morbidity were detected. Data are pooled from 4 independent experiments each with cohorts of 5 mice/group.

Supplementary Video Legends

Supplemental Video 1. Example of a control B-ALL cell undergoing transendothelial migration imaged by time-lapse confocal microscopy. Control ZsGreen+ B-ALL cells were flowed onto bEnd.3 endothelial cell monolayers. Using a spinning-disk confocal microscope, fluorescence and phase contrast time-lapse images were acquired for up to 30 min while the cells were maintained under a 2 dyne/cm^2 shear flow. Phase contrast (left), ZsGreen fluorescence (middle), and overlay (right) images are shown. This control B-ALL cell completes transendothelial migration as shown by the progressive disappearance of the white phase contrast ring around the B-ALL cell. Time is displayed as hrs:min:sec.

Supplemental Video 2. Example of an mDia1 KD B-ALL cell failing transendothelial migration imaged by time-lapse confocal microscopy. mDia1 KD ZsGreen+ B-ALL cells were flowed onto bEnd.3 endothelial cell monolayers. Using a spinning-disk confocal microscope, fluorescence and phase contrast time-lapse images were acquired for up to 30 min while the cells were maintained under a 2 dyne/cm^2 shear flow. Phase contrast (left), ZsGreen fluorescence (middle), and overlay (right) images are shown. This representative mDia1 KD B-ALL cell fails to complete transendothelial migration as shown by the persistence of the white phase contrast ring around the B-ALL cell. Time is displayed as hrs:min:sec.
